# Cosmeceutical Effects of *Ishige okamurae* Celluclast Extract

**DOI:** 10.3390/antiox11122442

**Published:** 2022-12-10

**Authors:** Fengqi Yang, Jimin Hyun, D. P. Nagahawatta, Young Min Kim, Moon-Soo Heo, You-Jin Jeon

**Affiliations:** 1 Department of Marine Life Sciences, Jeju National University, Jeju 63243, Republic of Korea; 2 Marine Science Institute, Jeju National University, Jeju 63333, Republic of Korea; 3 Aqua Green Technology Co., Ltd., Smart Bldg., Jeju Science Park, Cheomdan-ro, Jeju 63309, Republic of Korea

**Keywords:** *Ishige okamurae*, Celluclast, sulfated polysaccharides, cosmeceutical effect, antioxidant activity, anti-inflammatory activity, photoprotective effect

## Abstract

Sulfated polysaccharides extracted from brown algae are unique algal polysaccharides and potential ingredients in the cosmeceutical, functional food, and pharmaceutical industries. Therefore, the present study evaluated the cosmeceutical effects, including antioxidant, anti-wrinkle, anti-inflammation, and photoprotective activities, of *Ishige okamurae* Celluclast extract (IOC). The IOC was abundant in sulfated polysaccharides (48.47%), polysaccharides (44.33%), and fucose (43.50%). Moreover, the IOC effectively scavenged free radicals, and its anti-inflammatory properties were confirmed in lipopolysaccharide-induced RAW 264.7 macrophages; therefore, the IOC may produce auxiliary effects by inhibiting reactive oxygen species (ROS). In vitro (Vero cells) and in vivo (zebrafish) studies further confirmed that the IOC produced a protective effect against hydrogen-peroxide-induced oxidative stress in a dose-dependent manner. In addition, the IOC suppressed intracellular ROS and apoptosis and enhanced HO-1 and SOD-1 expression through transcriptional activation of Nrf2 and downregulation of Keap1 in HaCaT cells. Furthermore, the IOC exhibited a potent protective effect against ultraviolet-B-induced skin damage and photoaging. In conclusion, the IOC possesses antioxidant, anti-inflammatory, and photoprotective activities, and can, therefore, be utilized in the cosmeceutical and functional food industries.

## 1. Introduction

Reactive oxygen species (ROS), which are present in various forms, are physiological metabolites generated through oxygen metabolism under normal physiological conditions. Various factors, including ultraviolet-B (UVB) radiation, heavy metal ions, drugs, pollutants, and fine dust particles, promote ROS production [1,2,3]. However, ROS overproduction can interfere with the mitochondrial membrane potential and disrupt the cellular redox balance, resulting in the induction of oxidative stress and development of numerous diseases, including neurological disorders, cardiovascular disease, digestive diseases, and certain cancers [4,5]. As the skin is inevitably exposed to UVB rays in daily life, this can lead to oxidative damage of the skin tissues and cells. Excessive ROS accumulation leads to photoaging and inflammation of the human skin, eventually resulting in dermal malignancies [6,7]. Moreover, some cosmetics containing specific chemicals, such as heavy metals, steroids, hydroquinones, and nitrosamines, albeit effective in the short-term, can harm human health in the long-term [8]. Therefore, eco-friendly cosmetics containing natural ingredients must be developed. In recent years, cosmetic products with non-toxic and highly bioactive natural ingredients have attracted increasing research attention.

In this context, seaweeds have found wide pharmaceutical and industrial applications as natural sources of compounds because of their non-toxicity, easy cultivation, low cost, and presence of an array of active compounds, such as polysaccharides, proteins, minerals, vitamins, and pigments; therefore, seaweeds are the best candidates to replace synthetic compounds [9,10,11]. The majority of the algal species contain polysaccharides, polyphenols, and peptides, and most of these compounds exhibit biological functions, such as antioxidant, antimicrobial, antitumoral, anti-inflammatory, and UVB protective effects [12,13].

*Ishige okamurae* is one of the most common edible brown algae. It is widely distributed in the coastal areas of East Asia and contains abundant polyphenols, fucoxanthin, diphlorethohydroxycarmalol, and ishigoside bioactive compounds [7,14,15]. Many activities of *I. okamurae* have been investigated. For instance, the ethanolic extract of *I. okamurae* was effective against particulate matter-induced skin damage and produced antibacterial effects. In addition, the protective effects of the methanolic extract of *I. okamurae* against inflammatory myopathy as well as its anti-obesity, antioxidant, and anticancer activities have been reported [11,15,16,17]. Although there are many studies on *I. okamurae*, there is little information available on the cosmeceutical effects of *I. okamurae*, especially the Celluclast extract. A variety of extraction technologies are widely used, such as enzyme-assisted extraction, ultra-high-pressure extraction, microwave-assisted extraction technology, and supercritical fluid extraction. Compared with organic and water extraction, enzymatic extraction has a high catalytic efficiency and is more conducive to destroying the cell walls, which can not only improve the extraction yield but also show better biological activity, which is suitable for algae extraction [18,19]. In addition, enzyme-assisted extraction technology has mild reaction conditions and is safe. Compared with microwave and ultra-high-pressure extraction technologies, it has low equipment requirements, simple operation, is solvent-free, and has a moderate cost, so it is more suitable for food and cosmetic industrial production.

To this end, in the present study, Celluclast, a food-grade carbohydrase, was used to hydrolyze *I. okamurae*, and cosmeceutical effects of the *I. okamurae* Celluclast extract (IOC) were evaluated. Specifically, combining chemical technology, zebrafish, and cellular-level research, the antioxidant, anti-inflammatory, anti-wrinkle, and photoprotective activities of the IOC were evaluated to expand the applications of this brown alga in the cosmetic industry.

## 2. Materials and Methods

### 2.1. Materials

The collagenase from clostridium histolyticum, elastase from porcine pancreas, hyaluronidase, 2′,7′-dichlorodihydroflurescin diacetate (DCFH-DA), acridine orange, 3-(4-5-dimethyl-2yl)-2-5-diphynyltetrasolium bromide (MTT), dimethyl sulfoxide (DMSO), phosphate-buffered saline (PBS), standard gallic acid, 2,2′-azino-bis(3-ethylbenzothiazoline-6-sulphonic acid) (ABTS), H_2_O_2_, and the Human matrix metalloproteinases (MMP)-1, 2, 9 Enzyme-Linked Immunosorbent Assay (ELISA) kit were purchased from Sigma Co. (St. Louis, MO, USA). Penicillium/streptomycin (P/S), Dulbecco’s modified Eagle’s medium (DMEM), Roswell Park Memorial Institute-1640 (RPMI-1640) medium, trypsin-EDTA, and fetal bovine serum (FBS) were purchased from Gibco BRL (Life Technologies, Burlington, ON, Canada). The PGE2 kit used in the experiments was purchased from R&D Systems (Minneapolis, MN, USA). Anti-heme oxygenase 1 (HO-1), anti-superoxide dismutase 1 (SOD1), and anti-nuclear factor-erythroid 2-related factor-2 (Nrf2) antibodies were purchased from Cell Signaling Technology (Beverly, MA, USA). Kelch-like ECH-associated protein 1 (keap1) and β-actin antibody were purchased from Santa Cruz Biotechnology (Santa Cruz, CA, USA). All other chemicals used in this study were of analytical grade.

### 2.2. Sample Extraction

*I. okamurae* was harvested in April 2020 from Seongsan, Jeju Island, Republic of Korea. Following immersion in water for 24 h, the algae were washed three times with tap water to remove salt, sand, and debris. The algae were then freeze-dried and crushed. Lyophilized *I. okamurae* powder (100 g) was mixed with distilled water (1 L) at pH 4.5. Next, the mixture was extracted using Celluclast (1 g) in a continuous shaking incubator for 6 h at 50 °C. Subsequently, the enzyme was inactivated (100 °C) for 15 min. The pH of the extract was adjusted to 7.0, and the supernatant was separated using centrifugation (12,000× *g*, 15 min, 4 °C). The supernatant was then concentrated by evaporation and stored in a −80 °C deep freezer before freeze-drying. IOC powder was obtained by freeze-drying the mentioned supernatant.

### 2.3. Chemical Composition Analysis

Protein content was quantified using a commercial BCA protein assay kit (Thermo Scientific, Rockford, IL, USA). Sulfate content was analyzed using the BaCl_2_ gelatin method. The samples were hydrolyzed using 4 M trifluoroacetic acid for 5 h at 100 °C [20]. Gallic acid and glucose were used as the standards to evaluate the total phenolic and polysaccharide content, respectively [21,22]. The monosaccharide content of samples was determined using the Bio-LC (HPAEC-PAD system; Dionex, Sunnyvale, CA, USA) with the CarboPacTM PA1 column following pretreatment with 2 M trifluoroacetic acid. The samples were detected with the ED50 Dionex electrochemical detector using a standard containing fucose, arabinose, galactose, rhamnose, glucose, xylose, and fructose.

### 2.4. Radical Scavenging Assay

The radical scavenging activity was investigated using a previously published method [23]. In brief, ABTS (7 mM) and potassium persulfate (2.45 mM) were reacted in the dark for 12 h at 25 °C to generate the radicals. The ABTS and sample solutions were mixed and provided 10 min of incubation in the dark. The radical scavenging activity of the samples was evaluated at 734 nm.

### 2.5. Measurement of Enzyme Inhibitory Effects of IOC

#### 2.5.1. Collagenase Inhibitory Effect Assay

The collagenase inhibitory activity was estimated using 1 mg of Azocoll with 800 µL of 0.1 M Tris–HCl buffer (pH 7.0). An amount of 100 µL of 200 units·mL^−1^ of collagenase and 100 µL of the sample were added with 1 h incubation at 43 °C. Following incubation, the mixture was centrifuged (3000 rpm, 10 min) and the supernatant was measured at 540 nm [24]. 

#### 2.5.2. Elastase Inhibitory Effect Assay

The elastase inhibitory activity was estimated according to the previous method [25]. In brief, elastase was treated with a mixture of the sample and 1.015 mM N-succinyl-ala-ala-ala-ala-p-nitroanilide, and 10 min of incubation was provided at 25 °C. The microplate reader (BioTek Synergy HT, BioTek Instrument, Winooski, UT, USA) was utilized to measure the absorbance at 410 nm.

#### 2.5.3. Hyaluronidase (HAase) Inhibitory Assay

The HAase inhibitory activity was estimated following the previous method with slight modifications [26]. Briefly, the sample was mixed with HAase (10 mg·mL^−1^) dissolved in acetate buffer (pH 3.5) at a 1:1 ratio and provided 20 min of incubation at 37 °C. A further 20 min of incubation was provided with CaCl_2_ (12.5 mM). Then, hyaluronate was treated as a substrate and provided another 40 min of incubation. Finally, the mixture solution was incubated at 100 °C for 3 min after treating NaOH (0.4 N) and potassium tetraborate (0.4 M), and 180 µL of p-dimethylaminobenzaldehyde was added at 25 °C. The microplate reader (BioTek Synergy HT) was utilized to measure the absorbance at 540 nm.

### 2.6. Cell Viability Analysis

RAW 264.7, Monkey kidney fibroblasts (Vero), and human keratinocyte (HaCaT) cell lines were purchased from the Korean Cell Line Bank (KCLB, Seoul, Republic of Korea). Vero cells were maintained in RPMI-1640, and RAW 264.7 and HaCaT cells were cultured in DMEM under a controlled environment (37 °C, 5% CO_2_). All media contained 1% antibiotics and 10% fetal bovine serum. All cells were seeded at the density of 1 × 10^5^ cells·mL^−1^ in 96-well plates and cultured for 24 h. The cells were treated with varying concentrations of IOC (3.125, 6.25, 12.5, 25, 50, 100, and 200 µg·mL^−1^) and cell viability was evaluated using the MTT assay.

### 2.7. Effect of the IOC on H_2_O_2_-Induced Intracellular ROS Production and Cell Viability

After 24 h of Vero cells seeding, different concentrations of the IOC (3.125–200 µg·mL^−1^) were treated, incubated for 24 h, and then co-treated with hydrogen peroxide (600 µM). The cytoprotective effect of the IOC against H_2_O_2_-induced cell damage was assessed using cell viability. Cells were incubated with H_2_O_2_ for 1 h, and then 10 µL of DCFH-DA (25 µg·mL^−1^) was added and incubated for another 10 min. Excitation at 485 nm and emission at 530 nm were measured using a microplate reader (BioTek Synergy HT) [27].

### 2.8. Effect of the IOC NO and PGE2 Generation on Lipopolysaccharide (LPS)-Induced RAW 264.7 Macrophages

To evaluate the anti-inflammatory effect of the IOC, RAW 264.7 cells were treated with IOC for 1 h, following co-treatment with 1 µg·mL^−1^ of LPS for 23 h. Cell viability was assessed using the MTT assay. The supernatant was collected. NO and PGE2 production were analyzed using the Griess reagent and commercial ELISA kit, respectively [25].

### 2.9. Cytoprotective Activity of the IOC on UVB-Induced HaCaT Cells

A UVB meter containing a fluorescent bulb (wavelength 315–280 nm, peak 313 nm) was used to irradiate (30 mJ·cm^−2^) HaCaT cells to induce photodamage. Serum-free DMEM medium was used for the incubation of cells until analysis. The effect of the IOC (3.125–200 μg·mL^−1^) on UVB-induced photodamage was determined based on the measurement of intracellular ROS levels, apoptotic body formation, and UVB-irradiated HaCaT cell viability using the DCFH-DA assay, Hoechst 33342 staining, and MTT assay, respectively [24,28,29].

### 2.10. Western Blotting

The effects of the IOC on the expression of SOD-1, Nrf2, keap1, and HO-1 were assessed using Western blotting. IOC-treated and UVB-irradiated HaCaT cells were harvested and lysed. The BCA protein assay was used to quantify protein content in the lysate and it was subjected to 7.5% sodium dodecyl sulfate (SDS)–polyacrylamide gel electrophoresis (PAGE). The proteins were transferred onto nitrocellulose membranes blocked with 5% skim milk at 27 °C. Overnight incubation of membranes with primary antibodies at 4 °C was performed following incubation with secondary antibodies (Santa Cruz Biotechnology, Paso Robles, CA, USA) for 2 h at room temperature. Bands were detected using an ECL Western blotting detection kit and photographed using the FUSION SOLO Vilber Lourmat system (Paris, France).

### 2.11. Determination of Metalloproteinase (MMPs) Expression Levels Using ELISA

HaCaT cells were incubated with the IOC (50, 100, and 200 μg·mL^−1^) for 2 h and washed with PBS before UVB irradiation (30 mJ·cm^−2^). Following UVB irradiation, the cells were incubated with serum-free DMEM for 24 h. Culture media were collected, and MMP expression levels were quantified using a commercial ELISA kit according to the manufacturer’s instructions.

### 2.12. Origin and Maintenance of Zebrafish

Adult zebrafish were purchased from the commercial market (Jeju Aquarium, Republic of Korea). The zebrafish were separately maintained in 3 L tanks under a 14 h light/10 h dark cycle at 28.5 °C and fed twice daily (Tetra GmgH D-49304, Melle, Germany). In the morning, embryos were obtained from natural spawning through the breeding of two males and one female.

### 2.13. Measurement of Heart Beating Rate, Survival Rate, ROS Generation, and Cell Death in Zebrafish Embryos

The protective effect of IOC against H_2_O_2_ was investigated using approximately 7–9 h post-fertilization (hpf) embryos by treating IOC (50, 100, and 200 μg·mL^−1^); controls were untreated. Following treatment for 1 h, the embryos were stimulated with H_2_O_2_ (5 mM), and the plate was incubated for 3 dpf. The survival rate and heart beating rate were counted according to the previously optimized method [30]. Acridine orange and DCFH-DA were utilized to evaluate the cell death and intracellular ROS generation, respectively [31]. Zebrafish larvae were photographed using a fluorescence microscope (CoolSNAP-Pro Color Digital Camera; Olympus, Japan) and the fluorescence intensity was quantified using ImageJ software (version 1.50i, NIH, USA).

### 2.14. Statistical Analysis

The data were analyzed using one-way ANOVA and Dunnett’s multiple comparisons test by GraphPad Prism 8 (GraphPad Software, Inc., San Diego, CA, USA). All the experiments were repeated in triplicate. All data are expressed as the mean values with the standard error of the mean (SEM). *p* < 0.05 was considered significantly different.

## 3. Results

### 3.1. Chemical Composition of IOC

Chemical composition was investigated after *I. okamurae* was hydrolyzed using Cellucalst. As shown in Table 1, the IOC contained 5.32 ± 0.43% protein, 3.82 ± 0.31% phenolics, 4.14 ± 0.12% sulfates, and 44.33 ± 0.65% polysaccharides. Overall, the IOC contained 48.47% sulfated polysaccharides. Furthermore, the monosaccharide content of the IOC was determined. The IOC contained 43.50% fucose, 36.39% glucose, 7.96% galactose, 11.74% xylose, 0.17% rhamnose, and 0.25% arabinose.

### 3.2. Enzyme Inhibitory Effects of the IOC

**Figure 1:**
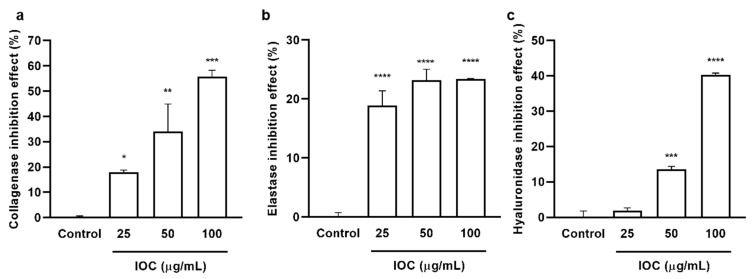
IOC inhibits commercial collagenase, elastase, and hyaluronidase: (**a**) collagenase inhibitory activity of IOC; (**b**) elastase inhibitory activity of IOC; (**c**) hyaluronidase inhibitory activity of IOC. All experiments were triplicated, and data are expressed as the mean ± SEM. * *p* < 0.05, ** *p* < 0.01, *** *p* < 0.001, and **** *p* < 0.0001 as compared to control group.

The inhibitory effects of the IOC on commercial collagenase, elastase, and HAase were examined, and the results are presented in Figure 1. The IOC significantly and dose-dependently increased collagenase and HAase inhibitory rates. The elastase inhibition rate did not differ among the concentration groups but was significantly increased relative to that in the control sample. At the IOC concentrations of 25, 50, and 100 µg·mL^−1^, the inhibitory rates were 18.01%, 34.11%, and 55.62% against collagenase; 18.85%, 23.20%, and 23.43% against elastase; 1.83%, 13.56%, and 40.23% against HAase, respectively. Therefore, the IOC may produce anti-wrinkle effects through the inhibition of collagenase, elastase, and HAase.

### 3.3. Antioxidant Effect of the IOC

#### 3.3.1. Free Radical Scavenging Activities of IOC

**Figure 2:**
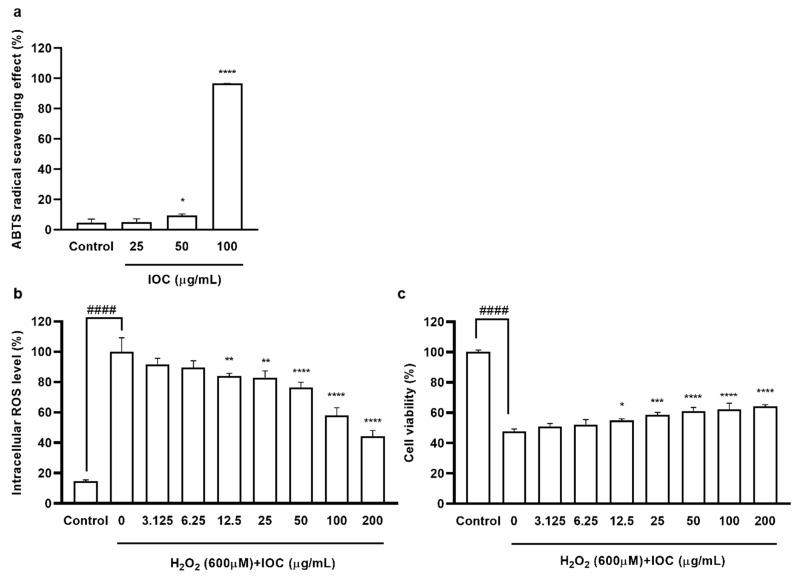
(**a**) ABTS free radical scavenging activity of IOC; (**b**) the intracellular ROS scavenging effect of IOC during H_2_O_2_ stimulated oxidative stress in Vero cells; (**c**) the protective effect of IOC against H_2_O_2_ stimulated cell death in Vero cells. All experiments were triplicated, and data are expressed as the mean ± SEM. In ABTS radical scavenging activity results: * *p* < 0.05 and **** *p* < 0.0001 as compared to control group. In intracellular ROS level and cell viability results: * *p* < 0.05, ** *p* < 0.01, *** *p* < 0.001, and **** *p* < 0.0001 as compared to the H_2_O_2_-treated group, and ^####^ *p* < 0.0001 as compared to the control group.

The free radical scavenging activity of the IOC was assessed using the ABTS assay. The activity of the IOC against ABTS radical cations was measured using concentrations ranging between 25 and 100 µg·mL^−1^. The percent radical scavenging activity at different concentrations of the extract is shown in Figure 2a. Significant scavenging activity was noted at 50 and 100 µg·mL^−1^ concentrations compared with the control value. In particular, the ABTS radical scavenging activity exceeded 96% at the concentration of 100 µg·mL^−1^.

#### 3.3.2. Effect of IOC on H_2_O_2_-Induced Intracellular ROS Generation and Cytotoxicity in Vero Cells

Chemical methods revealed that the IOC possesses antioxidant and anti-wrinkle activities. To further detect IOC activities, in vitro experiments were performed, and the sample concentration range was expanded. Specifically, seven concentrations (3.125–200 µg·mL^−1^) were tested to determine cytotoxicity. Cell viability did not differ significantly among the different concentrations. Hence, these concentrations (3.125–200 µg·mL^−1^) were used in further experiments. The intracellular ROS levels and viability of Vero cells following H_2_O_2_ stimulation are shown in Figure 2. There were no significant differences in intracellular ROS levels and cell viability between the 3.125 and 6.25 µg·mL^−1^ concentration groups; however, 12.5 to 200 µg·mL^−1^ concentration groups significantly differed from the control group and the significance of differences was dose-dependent. ROS generation tended to decrease (Figure 2b), whereas cell viability tended to increase in 12.5–200 µg·mL^−1^ concentration groups compared with values in the control group (Figure 2c).

### 3.4. Effect of the IOC on NO and PGE2 Production in LPS-Induced RAW 264.7 Cells

**Figure 3:**
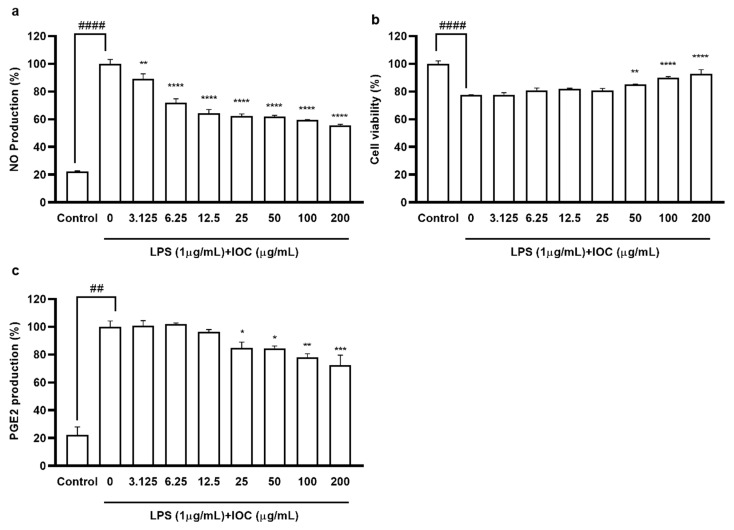
Effect of IOC on LPS-induced NO and PGE2 release by RAW 264.7 cells. (**a**) NO production inhibitory effect of IOC; (**b**) cytoprotective; (**c**) the level of PGE2. All experiments were triplicated, and data are expressed as the mean ± SEM. * *p* < 0.05, ** *p* < 0.01, *** *p* < 0.001, and **** *p* < 0.0001 as compared to the LPS-treated group; ^##^ *p* < 0.01 and ^####^ *p* < 0.0001 as compared to the control group.

To evaluate the anti-inflammatory effects of the IOC, we assessed the suppression of NO and PGE2 production in LPS-induced RAW 264.7 macrophages. We selected the concentration of 3.125–200 µg·mL^−1^ based on its non-cytotoxicity to macrophages (data not shown). As shown in Figure 3, when cells were exposed to LPS, NO and PGE2 production was significantly increased but cell viability was reduced compared with the control values. NO secretion was downregulated following IOC pretreatment in a dose-dependent manner, and cell viability increased significantly following treatment with 50, 100, and 200 µg·mL^−1^ of the IOC. In addition, the IOC (25, 50, 100, and 200 µg·mL^−1^) inhibited LPS-induced PGE2 production in RAW 264.7 cells.

### 3.5. Effects of the IOC on Intracellular ROS Levels and Cell Viability in UVB-Induced HaCaT Cells

**Figure 4:**
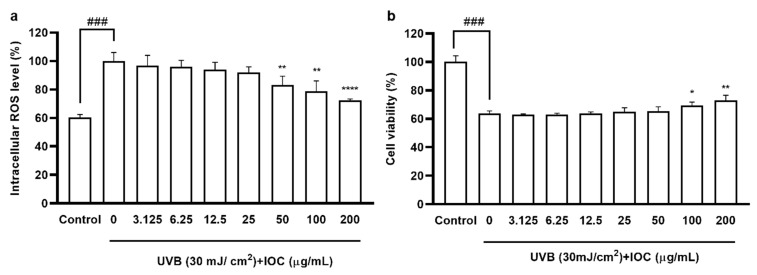
IOC inhibits intracellular ROS levels in UVB-irradiated HaCaT cells. (**a**) Intracellular ROS scavenging effect of IOC in UVB-induced HaCaT cells; (**b**) protective effects of IOC against UVB-irradiated HaCaT cells damage. All experiments were triplicated, and data are expressed as the mean ± SEM. * *p* < 0.05, ** *p* < 0.01, and **** *p* < 0.0001 as compared to the UVB-irradiated group, and ^###^ *p* < 0.001 as compared to the control group.

According to the cytotoxicity results, there were no significant differences in cell viability among 3.125 to 200 µg·mL^−1^ concentration groups, confirming that the IOC was non-toxic to HaCaT cells in this range (data not shown); thus, concentration ranges were selected for subsequent experiments. As shown in Figure 4a, UVB exposure increased intracellular ROS levels. However, treatment with the IOC reversed this effect in a dose-dependent manner. The cell viability of UVB-induced HaCaT cells was drastically reduced. However, this effect was restored following treatment with 100 and 200 µg·mL^−1^ of the IOC (Figure 4b).

### 3.6. Effect of the IOC against UVB-Induced Apoptosis

**Figure 5:**
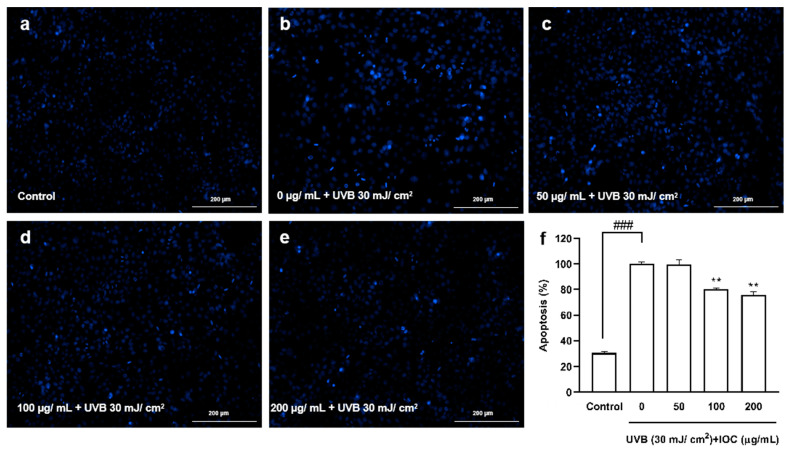
The protective effects of IOC against UVB-induced apoptosis in HaCaT cells. (**a**) Non-UVB-irradiated cells; (**b**) 30 mJ·cm^−2^ UVB-irradiated cells; (**c**) cells treated with 50 µg·mL^−1^ of IOC and UVB; (**d**) cells treated with 100 µg·mL^−1^ of IOC and UVB; (**e**) cells treated with 200 µg·mL^−1^ of IOC and UVB; (**f**) quantification of apoptotic cells. ** *p* < 0.01 as compared to the UVB-irradiated group and ^###^ *p* < 0.001 as compared to the control group.

Based on the above experimental results, higher concentrations of the IOC were found to present stronger activity, especially the concentrations from 50 to 200 µg·mL^−1^, which were significantly different from the other groups. Therefore, a concentration range of 50–200 µg·mL^−1^ was selected for subsequent experiments to verify the activity of the IOC. Figure 5 shows the protective effects of the IOC against UVB-induced apoptosis. Cells in the control group, which were untreated and not exposed to UVB, contained intact nuclei, whereas cells in the negative control group, which were untreated but exposed to UVB, showed significantly fragmented nuclei. However, nuclear fragmentation was reduced in the IOC treatment groups, especially in the 100 µg·mL^−1^ and 200 µg·mL^−1^ treatment groups. Therefore, the IOC suppresses apoptosis following UVB irradiation, protecting the HaCaT cells from UVB-induced photodamage.

### 3.7. Effect of the IOC on UVB-Induced Antioxidant Enzyme Expression through Nrf2 Activation in HaCaT Cells

**Figure 6:**
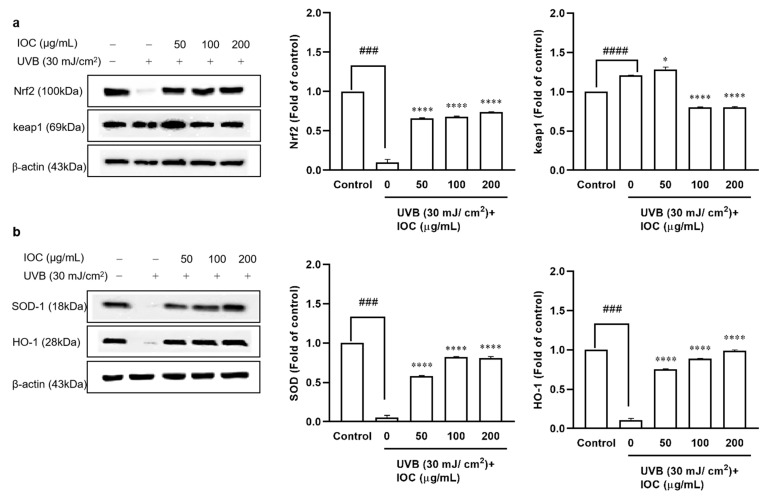
Effect of IOC on antioxidant-related protein in UVB-irradiated HaCaT cells. (**a**) Protein levels of Nrf2 and Keap1; (**b**) protein levels of SOD-1 and HO-1. β-actin was used as internal control. Quantification was assisted with the ImageJ software. Results are represented as mean ± SEM. * *p* < 0.05 and **** *p* < 0.0001 as compared to the UVB-irradiated group; ^###^ *p* < 0.001 and ^####^ *p* < 0.0001 as compared to the control group.

The Nrf2–Keap1 pathway plays pivotal roles in regulating the induction of antioxidant enzymes. To evaluate the effects of the IOC on UVB-irradiation-induced antioxidant enzyme expression, Western blotting was performed to analyze protein levels. Nrf2, SOD-1, and HO-1 expression was significantly inhibited following UVB irradiation. However, co-treatment with the IOC significantly restored the expression of these antioxidant enzymes in a dose-dependent manner (Figure 6a,b). Treatment with the IOC activated the Nrf2–Keap1 pathway, enhanced Nrf2 expression, and suppressed Keap1 expression. While UVB irradiation upregulated Keap1 expression, high concentrations of IOC downregulated it (Figure 6a). Therefore, the IOC inhibited intracellular ROS generation by promoting the activation of Nrf2 and inducing the expression of antioxidant enzymes, such as SOD-1 and HO-1.

### 3.8. Effects of IOC on MMPs Expression in UVB-Induced HaCaT Cells

**Figure 7:**
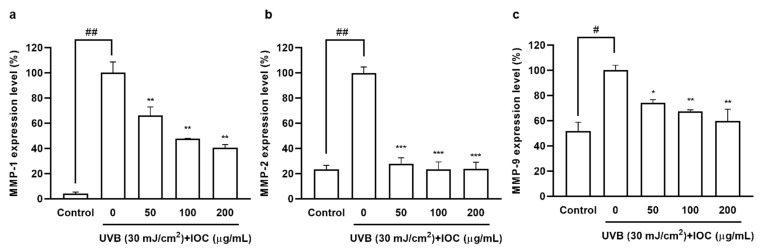
IOC reduces MMPs expression in UVB-irradiated HaCaT cells. (**a**) MMP-1 expression level in UVB-irradiated HaCaT cells; (**b**) MMP-2 expression level in UVB-irradiated HaCaT cells; (**c**) MMP-9 expression level in UVB-irradiated HaCaT cells. All experiments were triplicated, and data are expressed as the mean ± SEM. * *p* < 0.05, ** *p* < 0.01, and *** *p* < 0.001 as compared to the UVB-irradiated group; ^#^ *p* < 0.05 and ^##^ *p* < 0.01 as compared to the control group.

Collagen acts as an important structural support system providing strength and elasticity to the skin. UVB exposure activates MMPs secretion, leading to collagen degradation, which is a hallmark of skin aging [32]. As shown in Figure 7, UVB irradiation significantly increased MMP1, 2, and 9 secretion, However, MMPs’ expression decreased in a dose-dependent manner in IOC-pretreated HaCaT cells. Therefore, the IOC inhibited MMPs expression, thereby playing an anti-wrinkle role, which is important to prevent photoaging.

### 3.9. Effects of the IOC on H_2_O_2_-Induced Oxidative Stress in Zebrafish

**Figure 8:**
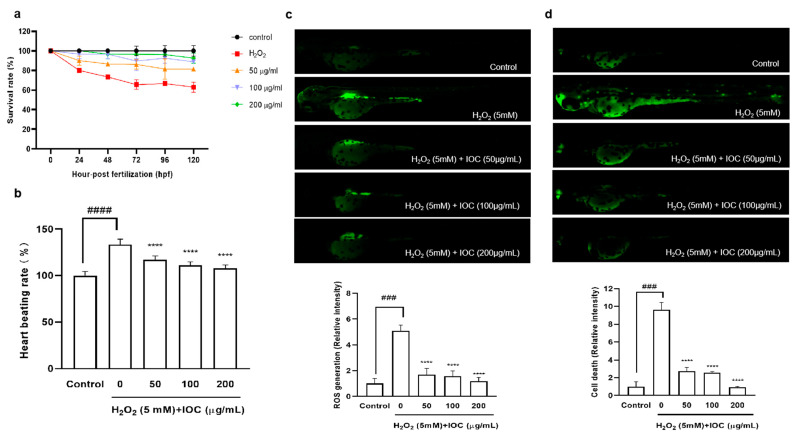
IOC depresses H_2_O_2_-induced oxidative damage in vivo in zebrafish. (**a**) Survival rate; (**b**) heart beating rate of both atrium and ventricle; (**c**) intracellular ROS generation; (**d**) cell death. All experiments were triplicated, and data are expressed as the mean ± SEM. **** *p* < 0.0001 as compared to the H_2_O_2_-induced group, ^###^ *p* < 0.001 and ^####^ *p* < 0.0001 as compared to the control group.

A zebrafish embryonic model was used to determine the effect of the IOC on H_2_O_2_-induced oxidative stress in vivo. The survival rate, heart beating rate, ROS generation, and cell death of zebrafish embryos were investigated. Figure 8 illustrated that the intracellular ROS and cell death decreased in a dose-dependent manner following treatment with the IOC. Additionally, compared with the control group, it was found that the survival rate was significantly decreased when stimulated with H_2_O_2_. However, this survival rate increased after treatment with 100 and 200 µg·mL^−1^ of the IOC. Furthermore, the heart beating rate of zebrafish was decreased in a dose-dependent manner in IOC-treated groups (50–200 µg·mL^−1^) compared with that in the H_2_O_2_-induced group. These observations confirmed that the IOC may produce protective effects against H_2_O_2_-induced oxidative stress.

## 4. Discussion

Among many biological processes, inflammation, oxidation, and photodamage are linked to one another. UVB radiation from the sun can activate complex biochemical reactions that induce skin damage and aging. Simultaneously, excessive UVB irradiation decreases cellular antioxidant levels, resulting in ROS accumulation [33]. When the ROS concentration exceeds the basal level, cellular defenses against oxidative stress are weakened, stimulating the expression of pro-inflammatory factors and MMPs, and leading to an inflammatory response and accelerating skin aging [34,35]. 

*I. okamurae* is an edible brown alga belonging to the Ishige genus of the Ishigeaceae family. *I. okamurae* contains a number of bioactive compounds and shows various bioactivities. For instance, Kang et al. [11] reported that isophloroglucin A derived from *I. okamurae* shows anti-obesity activity. Moreover, fucoxanthin has been reported to inhibit LPS-induced inflammatory responses in RAW 264.7 cells through the suppression of NF-ĸB activation and MAPK phosphorylation [14]. However, the cosmeceutical effects of the sulfated polysaccharide extract from *I. okamurae* remained to be assessed. Therefore, we used different cell lines as well as in vitro and in vivo methods to explore the cosmeceutical effects of IOC from multiple perspectives, such as anti-inflammatory, antioxidant, and photoprotective effects.

The IOC contains abundant sulfated polysaccharides (48.47%), fucose (43.50%), and glucose (36.39%). The extraction of bioactive compounds from seaweeds is restricted by the complexity of their cell wall polysaccharides. As Celluclast can degrade the seaweed cell wall, enzyme-assisted extraction helps the release of biologically active substances and contributes to increasing the content of polysaccharides and fucose in the extract [19]. As shown in Table 1, carbohydrates were concentrated during the precipitation process, and high amounts of sulfated polysaccharides were confirmed, which contributed to the cosmeceutical effects. Previous in vitro and in vivo studies have shown that sulfated polysaccharide extracts from Hizikia fusiforme possess antioxidant, anti-inflammatory, and photoprotective activities [36]. Sanjeewa et al. [37] reported that fucoidan—a sulfated polysaccharide isolated from Sargassum horneri—produced anti-inflammatory effects in LPS-induced cells. Moreover, Kim et al. [38] demonstrated that polysaccharides isolated from Psidium guajava exhibited antioxidant effects; simultaneously, fucose, galactose, and rhamnose levels were increased in the sulfate polysaccharide fraction. Fucose, galactose, rhamnose, and arabinose are associated with antioxidant activity. Organic free radicals, such as DPPH, ABTS, and ORAC, are commonly used to assess antioxidant activity. Therefore, in the present study, the antioxidant activity of the IOC was evaluated using the ABTS assay. As expected, the IOC possessed potent free radical scavenging activity, particularly at the concentration of 100 µg·mL^−1^. The balance between enzymatic antioxidants and free radicals is important for effective intracellular oxidative stress relief. In the present study, a significant and dose-dependent decline in intracellular ROS levels was noted following IOC treatment. Oxidative stress affects cell viability. Abundant fucose in the IOC implies greater antioxidant potential; accordingly, increasing the IOC concentration produced a protective effect against H_2_O_2_-stimulated cell death in Vero cells. 

Inflammation is a necessary component of physiological defense processes and is a response to cellular damage caused by oxidative stress, radiation (e.g., UV radiation), and endotoxins (e.g., LPSs) [6]. LPS, a crucial cell wall component of Gram-negative bacteria, induces an inflammatory response in macrophages, promoting the production of inflammatory mediators, such as NO, TNF-α, PGE2, IL-6, and IL-1β [29]. Simultaneously, increased NO production promotes ROS generation, which, in turn, induces apoptosis. Oxidative stress and inflammatory responses are interrelated. Macrophage production of PGE2 and proinflammatory cytokines plays key roles in the inflammatory process. To confirm the cytotoxicity and anti-inflammatory activity of the IOC, the viability of LPS-induced RAW 264.7 macrophages and inhibition of the production of NO and PGE2, which are inflammatory response indicators, were confirmed. At all concentrations, the IOC suppressed NO secretion in LPS-stimulated RAW 264.7 cells, and PGE2 levels decreased significantly in a dose-dependent manner with increasing IOC concentrations. Similarly, Sanjeewa et al. [37] showed that fucoidan—a complex sulfated polysaccharide isolated from Sargassum horneri—exhibits potent anti-inflammatory activity by blocking the MAPK and NF-ĸB signaling pathways. In addition, Jayawardena et al. [39] demonstrated that the anti-inflammatory potential of fucoidan extracted from the brown alga Turbinaria ornata was assisted by enzymatic hydrolysis in both in vivo and in vitro models. 

UV radiation can cause photodamage, wrinkles, and skin diseases by inducing excessive production of intracellular ROS and stimulating the expression of matrix MMPs and pro-inflammatory cytokines [34,40]. Natural aging and photoaging are the major causes of skin aging, manifesting as dryness, hyperpigmentation, laxity, and wrinkles [41]. Additionally, UV-radiation-mediated ROS production has been shown to promote the expression of specific genes involved in signaling pathways, resulting in various physiological effects, such as inflammatory responses. Increased ROS and H_2_O_2_ concentrations activate the NF-κB pathway, thereby increasing PGE2 and cytokine levels [6,35]. Therefore, we examined the inhibitory effects of the IOC on wrinkling-related enzymes. The increased activity of skin enzymes, such as collagenase, elastase, and HAase, leads to the proteolysis of the extracellular matrix (ECM). Furthermore, upregulated expression of MMPs, particularly MMP-2 and MMP-9, can lead to ECM degradation. Another important collagenase is MMP-1, which mainly degrades type I and type III collagen [36]. Chronic UV exposure denatures collagen and enzymes in the dermis, and elastin and collagen degradation results in the loss of skin elasticity and reduction in skin thickness, respectively. These are the major reasons for skin aging and wrinkling [24,42,43]. Our results showed that the IOC produced inhibitory effects on collagenase, elastase, and HAase and dose-dependently downregulated the expression of MMP-1, MMP-2, and MMP-9. Therefore, the IOC may act as a potential anti-wrinkle agent by interrupting the degradation of these skin enzymes under UV exposure. HaCaT cells, which are immortalized human keratinocytes, are widely used to study epidermal homeostasis. Therefore, we investigated the photoprotective activity of the IOC following UVB stimulation of HaCaT cells. The protective effects of the IOC against UVB irradiation were observed through cell viability assays. Our experiments revealed strong photoprotective activity of the IOC. As such, IOC treatment delayed skin aging by downregulating ROS production and protecting against nuclear fragmentation in UVB-stimulated HaCaT cells.

Nearly 80% of ROS production is induced by UV radiation. Therefore, antioxidant substances are critical for inhibiting oxidative damage and protecting the skin from photodamage. Activation of the Nrf2–Keap1 signaling pathway maintains high levels of antioxidant enzymes, such as SOD-1 and HO-1, which play pivotal roles in protection against photoaging [44]. The IOC significantly enhanced the levels of SOD-1 and HO-1 antioxidants through upregulating Nrf2 expression and significantly downregulating Keap1 expression. Therefore, the IOC produced protective effects against UVB-induced oxidative damage and apoptosis in HaCaT cells, primarily through the activation of the Nrf2 signaling pathway. In a similar study, Oh et al. [45] showed that 3,5-dicaffeoyl-epi-quinic acid reduced oxidative stress and prevented photoaging through upregulation of the antioxidant enzyme transcription factor Nrf2. These results imply that the IOC can serve as an effective skin protective ingredient in cosmetics.

ROS plays a significant role in oxidative stress, causing the breakdown of DNA, proteins, cell membranes, and other constituents. The accumulation of molecular damage leads to apoptosis, necrosis, and death. H_2_O_2_ is associated with the formation of hydroxyl and singlet oxygen radicals, which can stimulate intracellular ROS production and cause cell damage and senescence [46,47]. As a result, antioxidative substances are critical for preventing oxidative stress. Antioxidants in cosmeceuticals play critical roles in suppressing and inhibiting oxidative damage reactions. Therefore, the in vivo antioxidant effects of the IOC were investigated in a zebrafish embryo model. Because of their suitability for studying human disease processes and development, zebrafish are considered a painless in vitro alternative, becoming one of the most widely used vertebrate models [48]. Following H_2_O_2_ stimulation, excess intracellular ROS are generated, leading to cell death. A dose-dependent reduction in ROS levels and remarkably reduced cell death rate were observed in zebrafish following treatment with the IOC. The survival rate of H_2_O_2_-stimulated zebrafish was significantly decreased; however, the rate increased following co-treatment with IOC. In addition, H_2_O_2_ stimulation caused heartbeat disorder in zebrafish; however, co-treatment with IOC effectively downregulated the heart rate of zebrafish. In summary, our in vitro and in vivo results indicate that the IOC possesses potent anti-inflammatory and antioxidant activities and presents photoprotective potential.

## 5. Conclusions

We investigated the effect of sulfated polysaccharides from Celluclast-assisted extract of the brown seaweed *I. okamurae* as a source of natural cosmetic ingredients. IOC produced protective effects against H_2_O_2_-induced oxidative stress both in vitro (Vero cells) and in vivo (zebrafish). Furthermore, IOC produced anti-inflammatory effects in RAW 264.7 macrophages, and IOC exhibited antioxidant, anti-wrinkle, and photoprotective effects by suppressing UVB-induced oxidative stress, activating the Nrf2–Keap1 signaling pathway, and reducing MMPs expression in HaCaT cells. Therefore, the IOC may be used as an effective ingredient in the functional food and cosmetic industries.

## Figures and Tables

**Table 1:** Chemical composition of IOC obtained from *I. okamurae*.

Sample		IOC
Protein content%		5.32 ± 0.43
Phenolic content%		3.82 ± 0.31
Sulfate content%		4.14 ± 0.12
Polysaccharide content%		44.33 ± 0.65
Sulfated polysaccharide%		48.47
Monosaccharide %	Fucose	43.50
	Glucose	36.39
	Galactose	7.96
	Xylose	11.74
	Rhamnose	0.17
	Arabinose	0.25

## Data Availability

The data are contained within the article.
